# Nutritional value and organoleptic assessment of traditionally smoked cheeses made from goat, sheep and cow’s milk

**DOI:** 10.1371/journal.pone.0254431

**Published:** 2021-07-22

**Authors:** Magda Filipczak-Fiutak, Agnieszka Pluta-Kubica, Jacek Domagała, Iwona Duda, Władysław Migdał

**Affiliations:** Department of Animal Product Processing, Faculty of Food Technology, University of Agriculture in Krakow, Krakow, Poland; Institute for Biological Research, University of Belgrade, SERBIA

## Abstract

The use of small ruminant milk for smoked cheese production makes it possible to incorporate valuable nutrients into the diet, especially as the consumption of unprocessed sheep or goat’s milk is low compared to that from cows. Smoking of food not only prolongs its shelf-life but also improves its flavour. Taking the fact that many consumers do not accept some organoleptic properties of milk from small ruminants into account, the aim of the study was to assess and compare the organoleptic and nutritional properties of traditionally smoked cheeses made from goat, sheep and cow’s milk. The analysed cheeses differed in terms of dry matter content and its components such as protein and fat. Their acidity was comparable, except for the sample made of raw goat’s milk, which was characterised by a relatively high pH value (6.12 ± 0.06). The highest content of CLA (2.30 ± 0.04%), as well as the highest share of unsaturated and polyunsaturated fatty acids, was determined in the cheese made from sheep’s milk. Moreover, the content of butyric and caproic free fatty acids in cheeses made from goat’s milk was found to be several times higher than in the other analysed cheeses. The organoleptic assessment did not reveal any significant differences between the cheeses produced at small, private farms and in industrial conditions, or between different types of cheese, regardless of the type of milk from which they were produced.

## Introduction

Worldwide, the most commonly consumed milk comes from cows– 85% of the world milk production is derived from cattle. However, in certain parts of the world, milk from other animal species also has a significant share in milk consumption. Except for bovine milk, 11% of the world production is from buffalo milk, followed by caprine and ovine milks– 12.3 and 1.7%, respectively [1–3]. In Poland, sheep’s milk is particularly considered a very important part of agriculture in mountain areas. Increased milk production by small ruminants, including sheep and goats, has been observed lately, and now, there is a need to search for new consumers or convince those who prefer to drink only cow’s milk [3, 4]. Whole, fresh goat and sheep milk production in Poland has grown in recent years. In 2016, it equalled 6,913 and 473 tonnes, respectively. In 2017, 7,363 tonnes of goat’s milk and 552 tonnes of sheep’s milk were produced, while in 2018–7,451 and 606 tonnes, respectively [5]. Goat’s milk is mostly used for cheese and fermented milk production, as well as for direct consumption. In contrast, sheep’s milk is almost exclusively utilised for cheese-making and is rarely consumed as a drink [6, 7].

The chemical composition of milk depends on the requirements of the offspring regarding a given animal species, as their nutritional requirements vary. Therefore, milk composition is highly dependent on animal species. Moreover, the chemical composition of milk varies over time and among animals of the same species. This depends on the following factors: the stage of lactation, season, environmental temperature, lactation efficiency, animal age and nutrition, genetic factors (species and breed), and diseases of the udder [2, 4, 6, 8]. Regardless of the species, milk is composed of such main components as: fat, protein, lactose and ash. The component of milk having major impact on its nutritional value and technological suitability is protein, composed mostly of casein (approximately 80%) and 20% of whey proteins. Casein is not a homogeneous protein. It is composed of 4 fractions: αs1-casein, αs2-casein, β-casein and κ-casein [2, 4, 9]. The ratio between the milk casein fractions depends on the ruminant species. Moreover, micelle size, hydration and mineralisation also differ. When compared to cow’s milk, that from sheep and goats varies more among individuals and breeds, especially in αs1-casein and αs2-casein content. This is caused by the occurrence of genetic polymorphism among milk proteins. These differences affect cheese production [10]. In addition, ovine and caprine milk casein micelles are characterised by a higher degree of mineralisation, being less heat stable and hydrated than bovine milk casein micelles [11]. β-caseins (multiphosphorylated β1- and β2-casein), which have an amino acid composition similar to bovine β-casein, constitute approximately 60% of all caseins of sheep’s milk [10]. Unlike bovine milk, the β-casein in ovine milk does not diffuse into the interior or join at the surface of the micelle under cold storage conditions. Therefore, the refrigerated storage of sheep’s milk should not adversely affect its rennet coagulation ability or reduce the firmness of the curd. This is especially important during the production of cheese from a mixture of evening and morning milks. Therefore, sheep’s milk curd is firmer and the rate of whey expulsion from the curd is slower than in cow and goat’s milk curds [3, 12].

Ovine milk contains almost twice as much protein as caprine and bovine milks. These proteins appear in molecular forms and are characterised by amino acid sequences thanks to which, they have high nutritional quality, as well as positive influence on digestibility and thermostability [4]. Goat and cow’s milks are different in terms of their constituents–the former has higher amounts of casein, medium-chain fatty acids, polyunsaturated fatty acids, conjugated linoleic acid (CLA), calcium, phosphorus, magnesium and copper [13]. Regarding nutritive quality, goat’s milk, in comparison to cow’s milk, demonstrates higher magnesium content, a 20 times higher concentration of taurine and greater concentration of vitamin D [2, 11, 14]. Additionally, sheep’s milk contains more calcium, magnesium, phosphorus, manganese, zinc, and copper than cow’s milk [9]. Among ruminant milks, ovine milk fat contains one of the highest levels of conjugated linoleic acid (CLA, 0.65 g/100 g of fatty acids), as well as a large amount of vaccenic acid (VA), being its physiological precursor [7].

Goat’s milk is considered to have higher digestibility and a lower incidence of allergic reaction than cow’s milk. Goat’s milk is less likely to cause an allergic reaction due to its lower or minimal level of αs1-casein fraction. The low content of this casein fraction is also significant, as it causes a more sensitive structure of goat casein, which enhances its susceptibility to digestive enzymes. In addition, a lower content of αs1-casein fraction reduces sensitivity to β-lactoglobulin, another allergenic protein which is resistant to gastric pepsin [9, 13, 15].

In many cases, an increase in the use of goat products is closely related to medical problems such as food allergies. Using goat’s milk instead of that from cows resolves 30–40% of these problems [16–18]. Due to the content of valuable nutrients in goat’s milk, it is an excellent substitute for bovine milk. According to many authors, it may be consumed without having any negative effects on people suffering from CMPA (cow’s milk protein allergy) [2, 16, 17].

Raw goat’s milk has a specific taste. Consequently, the organoleptic properties of goat’s milk and its dairy products significantly influence the consumer demand for these kinds of foods. As a result, consumers often choose other types of milk and dairy products, as the flavour of goat’s milk does not appeal to them [8]. The evaluation of rennet cheese made from different types of milk was presented by Garcia et al. [19], who carried out sensorial assessment of products manufactured using cow, goat and buffalo milk. All of the cheeses presented good acceptability indices for the evaluated attributes, except for the taste and aroma of goat’s milk cheese. Moreover, Queiroga et al. [20] observed that rennet cheese made from a mixture of goat and cow’s milk was more appreciated due to the less pronounced goat flavour than products made only from goat’s milk. Similar findings were reported by Dmytrów et al. [21] with regard to an acid curd cheese (*tvarog*) produced from goat’s milk and a mixture of cow and goat’s milk.

Smoking of food not only prolongs its shelf-life, but also improves its flavour [22]. Therefore, it is possible that the application of smoking may have a positive effect on the organoleptic quality of goat and sheep cheeses, the taste and smell of which are not acceptable to some consumers. Traditionally, in Poland, smoked cow’s milk cheeses are often manufactured in industrial conditions. On the other hand, based on the observation of commercially available food products on the Polish market, sheep and goat cheeses are mainly produced at small private farms. Moreover, these cheeses are valuable food products due to their high nutritional value. Thus, the aim of the study was to assess and compare the organoleptic and nutritional properties of traditionally smoked cheeses made from goat, sheep and cow’s milk.

## Materials and methods

### Cheese samples

**Fig 1:**
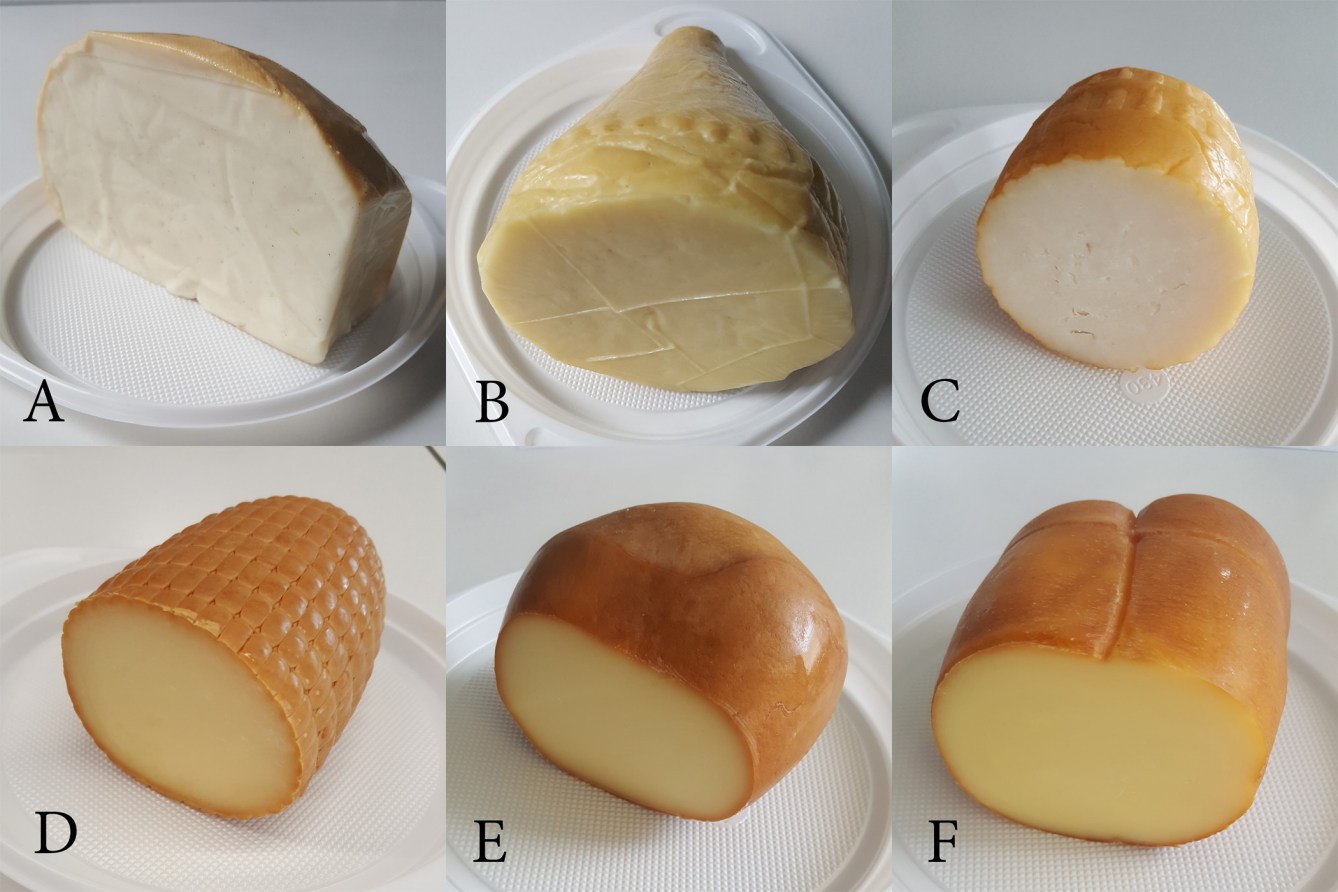
Appearance of the cheese samples.

The characteristics of the investigated rennet-curd cheeses are given in Table 1, and the appearance of the samples is presented in Fig 1. Samples B, D, E and F were pasta filata cheeses. All samples were traditionally smoked during the manufacturing process. Samples of cheeses were collected in 3 independent series (all with different batch numbers). All of the cheeses were manufactured in spring. The samples collected from small private farms were packed in aluminium foil, while the samples originating from industrial production were vacuum-packed in plastic packaging. All samples were transported in refrigerated conditions.

**Table 1:** Characterisation of the cheese samples.

Sample code	A	B	C	D	E	F
**Name**	*Bundz*	*Oscypek*	*Wołoski*	*Rolada sądecka*	*Sakwa*	*Gryficki*
**Type of milk**	raw caprine	raw ovine	raw caprine	pasteurised bovine	pasteurised bovine	pasteurised bovine
**Type of salting**	none	brining	brining	brining	brining	brining
**Smoking conditions**	warm smoke from beech and alder	warm smoke from spruce, pine, larch, alder and ash	warm smoke from beech, alder	warm smoke from beechalder	warm smoke from beech and oak	warm smoke from beech and oak
**Origin**	family-run ecological farm, Mszana, Podkarpacie region	agritourism farm, Leśnica, Małopolska region	family-run ecological farm, Mszana, Podkarpacie region	industrial conditions, NowySącz, Małopolska region	industrial conditions, Sanok, Podkarpacie	industrial conditions, Sanok, Podkarpacie

### Chemical composition and physicochemical properties

The content of water, protein, ash and NaCl were analysed according to AOAC [23–26], while fat was evaluated in accordance with ISO 3433:2008 [27]. Active acidity (pH) was obtained by conductometric measurements using a pH metre (CP-411, Elmetron, Poland). Water activity was measured using the LabMaster-aw (Novasina AG, Switzerland). All analyses were performed in triplicate.

### Amino acid composition

Amino acid content was determined in triplicate via the RP-HPLC method, using ACCQ Tag (Waters, USA). Approximately 30 mg of the sample were hydrolysed in 4 mL 6M HCl (POCH, Poland) containing 15 μL phenol (Sigma Aldrich, USA) at 110°C, for 24 hours and in a nitrogen atmosphere. The hydrolysates were filtered through a 0.45-μm syringe filter and evaporated just to dryness under steam from nitrogen. Derivatisation of samples was performed after appropriate dilution according to the procedure advised by Waters (USA), presented by Kabelová et al. [28] in detail. Furthermore, 10 μL of the diluted sample was mixed with a 70-μL borate buffer (pH range of 8.2–9.0) and 20 μL6-aminoquinolyl-N-hydroxysuccinimidyl carbamate (AQC) dissolved in acetonitrile (3 mg/mL). The same procedure was applied for derivatisation of amino acid standards (Waters, USA). Samples were analysed using the Dionex Ultimate 3000 (Thermo Scientific, country), equipped with an LPG-3400 SD gradient 4-component pump, WPS 3000 TSL autosampler and FLD-3400RSdetector with four absorption channels. The Nova–Pak C18 column (4 μm, 150 x 3.9 mm, Waters, USA) at 37°C was used. The elution programme was performed according to the procedure recommended by Waters (USA) and presented in detail by Kabelová et al. [28], using an acetate-phosphate buffer (pH 5.2) as eluent A and acetonitrile/water (60:40, Sigma Aldrich, country) as eluent B (1 mL/min). Extraction and emission wavelengths were 250 nm and 395 nm, respectively. A 1-point calibration scale was applied using a mixture of 17 amino acid analytical standards, each totalling 100 pmol/mL. The mixture of standards was produced by Waters (USA) and initially contained 2.5 μmol/mL of each amino acid. The results were compiled using Chromeleon 7.0 software.

### Fatty acid composition

Determination of fatty acids was performed in triplicate. Total lipids were extracted according to the Folch et al. [29] method, with modifications, while the esterification and determination of total fatty acid composition were carried out according to the de Man [30] method. The applied modifications were previously described by Domagała et al. [31]. A Supelcowax 10 column (with dimensions 30 m × 0.25 mm × 0.23 μm) and a gas chromatograph—Trace GC Ultra (Thermo Electron Corp., Waltham, USA), were used. The results were expressed as % of total peak area.

### Free volatile fatty acid (FVFA) content

Free volatile fatty acids: acetic, propionic, butyric, isovaleric, valeric and caproic—were determined in triplicate using the GC method according to Innocente et al. [32]. Crotonic acid (POCH S.A., Poland) was used as an internal standard. A Nukol column with the dimensions: 30 m × 0.53 mm × 0.5 μm (Supelco, USA), and the TRACE GC ULTRA gas chromatograph with FID (Thermo Electron Corporation, USA) were used. The chromatographic conditions of the GC were as follows: injector temperature—180°C, detector temperature—200°C, carrier gas—helium (LindeGazPolska, Poland), flow—25 cm^3^/min; oven temperature programme: initial temperature—80°C for 3 min, temperature ramp rate—8°C/min, final temperature—180°C maintained for 1 min.

### Texture profile analysis

Instrumental texture profile analysis (TPA) was performed using the Universal Texture Analyser TA-XTPlus (Stable Micro Systems, Surrey, UK), controlled by a computer. Samples were cut into cubes with a side length of 2 cm, and their temperature was adjusted to approximately 20°C. Compression at 60% deformation of the baseline sample height was performed with a test speed of 1 mm/s. The test was conducted using a compression platen of 10 cm in diameter (SMS P/100). Each sample was compressed in 2 consecutive compression cycles. The obtained diagrams of force-dependence on time were analysed using Texture Expert for Windows v. 1.05 (Stable Micro Systems, Surrey, UK). Each cheese was analysed 4 times.

### Organoleptic assessment

Organoleptic assessment of cheese was carried out using a 5-point scale. The following quality properties were evaluated (1—the worst; 5—the best): colour of the rind, colour of the cheese body, appearance of the cross section of cheese, smell, consistency and taste. The proper indices of significance were ascribed as follows: 0.15, 0.15, 0.10, 0.20, 0.15 and 0.25, respectively. The overall quality was calculated. A trained panel consisting of 8 persons, whose sensory sensitivity was proved, completed the evaluation. The panellists were tested for ageusia and anosmia, and taste as well as smell detection thresholds. They were instructed on the process of evaluating the different sensory attributes.

### Statistical analysis

The obtained results were statistically analysed using Statistica version 13.3 (TIBCO Software Inc., Palo Alto, CA, USA). Means and standard deviations were calculated. Furthermore, the Shapiro-Wilk and Levene’s tests were performed. One-way ANOVA was employed and the significance of differences between the means was established using Tukey’s test. If any variables did not meet the assumptions of the analysis of variance, non-parametric one-way ANOVA (Kruskal-Wallis test) and multiple comparisons on ranks of several independent samples were performed. Moreover, the results of organoleptic evaluation that appeared to be ordinal, were statistically analysed using the Kruskal-Wallis test by ranks. Additionally, Pearson’s correlation coefficients between the water activity and content of water and NaCl were calculated.

## Results and discussion

### Chemical composition and physicochemical properties

The results of chemical composition and physicochemical properties concerning the analysed cheeses are presented in Table 2. The highest water content was determined in A (51.57 ± 0.06%), while the lowest–in B (39.03 ± 0.31%). According to Council Regulation (EC) No 510/2006 [33], the dry matter in *oscypek* (B) should be not less than 56%, while fat in dry matter content–no less than 38%. The analysed cheese met both requirements. Among the analysed cheeses, 5 of them were characterised by similar fat in dry matter content. This characteristic was the lowest in C (27.37 ± 1.05%). However, C contained the highest amount of protein (32.97 ± 0.06%) and ash (6.86 ± 0.04%). Unfortunately, it also contained the highest amount of sodium chloride (2.57 ± 0.06%).

**Table 2:** Properties of cheeses.

Compound or feature	A	B	C	D	E	F
**Water [%]**	51.57^d^ ± 0.06	39.03^a^ ± 0.31	43.30^b^ ± 0.40	44.90^c^ ± 0.30	43.70^b^ ± 0.00	43.30^b^ ± 0.10
**Fat [%]**	19.77^b^ ± 0.25	27.59^e^ ± 0.37	15.50^a^ ± 0.50	23.00^c^ ± 0.00	27.25^e^ ± 0.25	24.75^d^ ± 0.25
**Fat [% of dry matter]**	40.80^b^ ± 0.60	45.53^d^ ± 0.65	27.37^a^ ± 1.05	41.70^b^ ± 0.20	48.37^e^ ± 0.45	43.67^c^ ± 0.35
**Protein [%]**	22.73^a^ ± 0.15	28.37^a^^b^ ± 0.15	32.97^b^ ± 0.06	29.43^a^^b^ ± 0.15	25.10^a^^b^ ± 0.10	27.87^a^^b^ ± 0.15
**Ash [%]**	4.16^d^ ± 0.01	4.06^c^ ± 0.04	6.86^e^ ± 0.04	3.68^a^ ± 0.03	3.88^b^ ± 0.01	4.00^c^ ± 0.04
**NaCl [%]**	1.80^a^^b^ ± 0.00	1.70^a^^b^ ± 0.00	2.57^b^ ± 0.06	1.37^a^ ± 0.06	1.77^a^^b^ ± 0.06	1.60^a^^b^ ± 0.00
**pH**	5.52^d^ ± 0.03	5.18^b^ ± 0.03	6.12^e^ ± 0.06	5.06^a^ ± 0.05	5.34^c^ ± 0.05	5.31^c^ ± 0.04
**Aw**	0.956^e^ ± 0.001	0.938^a^ ± 0.001	0.941^b^ ± 0.001	0.953^d^ ± 0.001	0.950^c^ ± 0.001	0.950^c^ ± 0.001

$\bar{x}$ ± sd; mean values ± standard deviation

a-e–statistically significant differences between means (*p*≤0.05) are marked by different letters in rows

Acidity of the analysed samples was typical for rennet-curd cheeses (pH ranging from 5.06 to 5.52), except for sample C, which was characterised by a relatively high pH value (6.12 ± 0.06). An increase in pH may occur during the storage of cheeses as a result of casein proteolysis [21]. According to Van Nieuwenhove et al. [34], the pH of cow and goat’s milk rennet-curd cheeses is similar. Fangmeier et al. [35] came to the same conclusions about pH regarding cow and goat’s milk cream cheeses.

The lowest water activity was determined in sample B, while the highest–in A. This characteristic depended on water and NaCl content. Water activity positively correlated with the amount of water (r = 0.82), and negatively with the amount of NaCl (r = -0.50). The correlations were statistically significant (*p*≤0.05).

The basic chemical composition of cheeses, apart from the main factor, i.e. the type of milk, is also influenced by production season and the length of smoking. In addition, the use or absence of pasteurisation during production affects this characteristic [36].

### Amino acid composition

Amino acid profiles of the analysed cheeses are presented in Table 3, and a sample chromatogram is shown in S1 Fig. Significant differences were observed in all amino acid contents except cysteine. Glutamic acid was a dominant amino acid present in all cheeses. Generally, most often, the highest content of individual amino acids was found in C, and the lowest—in A. This was probably due to the fact that C and A contained the highest and lowest amounts of protein, respectively (Table 2). Moreover, B, E and F were the most similar in terms of amino acid content.

**Table 3:** Amino acid profile of cheeses.

Amino acids [g /100g]	A	B	C	D	E	F
**Asp**	1.39^b^ ± 0.06	1.97^e^ ± 0.03	1.73^d^ ± 0.04	2.11^f^ ± 0.02	1.22^a^ ± 0.02	1.63^c^ ± 0.03
**Ser**	1.16^a^ ± 0.01	1.56^a^^b^ ± 0.03	1.71^b^ ± 0.01	1.64^a^^b^ ± 0.02	1.59^a^^b^ ± 0.01	1.49^a^^b^ ± 0.02
**Glu**	4.24^a^ ± 0.01	5.57^a^^b^ ± 0.12	6.52^b^ ± 0.02	6.22^a^^b^ ± 0.01	5.43^a^^b^ ± 0.04	5.48^a^^b^ ± 0.02
**Gly**	0.35^a^ ± 0.00	0.50^a^^b^ ± 0.01	0.48^a^^b^ ± 0.00	0.59^b^ ± 0.01	0.54^a^^b^ ± 0.00	0.50^a^^b^ ± 0.02
**His**	0.61^a^ ± 0.01	0.76^a^^b^ ± 0.01	0.96^b^ ± 0.01	0.81^a^^b^ ± 0.01	0.76^a^^b^ ± 0.01	0.77^a^^b^ ± 0.02
**Arg**	0.79^a^ ± 0.02	1.19^d^ ± 0.08	1.09^b^^c^^d^ ± 0.03	1.14^c^^d^ ± 0.01	1.05^b^^c^ ± 0.05	1.01^b^ ± 0.02
**Thr**	1.15^a^^b^ ± 0.03	1.22^a^^b^ ± 0.04	1.64^b^ ± 0.02	1.05^a^ ± 0.05	1.24^a^^b^ ± 0.01	1.24^a^^b^ ± 0.02
**Ala**	0.59^a^ ± 0.04	0.86^b^^c^ ± 0.04	0.77^b^ ± 0.07	0.94^c^^d^ ± 0.00	0.98^d^ ± 0.03	0.80^b^ ± 0.01
**Pro**	2.72^a^ ± 0.03	3.09^a^^b^ ± 0.09	4.16^b^ ± 0.03	3.25^a^^b^ ± 0.06	3.07^a^^b^ ± 0.01	3.20^a^^b^ ± 0.02
**Tyr**	0.55^a^ ± 0.01	0.77^a^^b^ ± 0.02	0.81^a^^b^ ± 0.00	0.92^b^ ± 0.00	0.88^a^^b^ ± 0.01	0.79^a^^b^ ± 0.02
**Val**	1.82^a^ ± 0.03	1.93^a^^b^ ± 0.07	2.70^b^ ± 0.02	2.14^a^^b^ ± 0.01	1.94^a^^b^ ± 0.05	2.09^a^^b^ ± 0.02
**Met**	0.75^a^ ± 0.03	0.85^b^ ± 0.03	0.92^c^ ± 0.01	0.88^b^^c^ ± 0.02	0.87^b^^c^ ± 0.02	0.84^b^ ± 0.03
**Cys**	0.09 ± 0.01	0.10 ± 0.00	0.08 ± 0.00	0.09 ± 0.00	0.49 ± 0.41	0.09 ± 0.01
**Lys**	1.90^a^ ± 0.03	2.47^a^^b^ ± 0.10	2.66^b^ ± 0.01	2.61^a^^b^ ± 0.06	2.30^a^^b^ ± 0.05	2.32^a^^b^ ± 0.03
**Ile**	1.21^a^ ± 0.02	1.41^b^ ± 0.05	1.71^d^ ± 0.01	1.56^c^ ± 0.00	1.43^b^ ± 0.04	1.45^b^ ± 0.02
**Leu**	2.31^a^ ± 0.03	2.81^a^^b^ ± 0.03	3.38^b^ ± 0.07	2.98^a^^b^ ± 0.03	2.83^a^^b^ ± 0.05	2.81^a^^b^ ± 0.03
**Phe**	1.17^a^ ± 0.02	1.39^a^^b^ ± 0.02	1.77^b^ ± 0.03	1.48^a^^b^ ± 0.01	1.46^a^^b^ ± 0.02	1.42^a^^b^ ± 0.02

$\bar{x}$ ± sd; mean values ± standard deviation

a-f–statistically significant differences between means (*p*≤0.05) are marked by different letters in rows

Goat’s milk is characterised by higher levels of 6 out of the 10 essential amino acids (threonine, isoleucine, lysine, cystine, tyrosine, valine) compared to cow’s milk. This was in agreement with our results. C (a goat’s milk cheese) was characterised by the highest content of the 4 above-mentioned amino acids–Thr, Val, Lys and Ile. Sheep’s milk is also richer in valine and lysine than cow’s milk. Moreover, it contains more serine, alanine and histidine [3, 37]. Their exact metabolic effect has not been extensively studied, however, some authors claim that their presence and amount may be an explanation for the beneficial effect of goat’s milk on human nutrition [16]. Furthermore, during a study on rats presenting malabsorption syndromes, it was found that goat’s milk improves the intestinal absorption of copper. This was explained by the higher content of cysteine (derived from cystine) in goat’s milk in comparison to cow’s milk (83 mg and 28 mg/100 g, respectively) [38].

### Fatty acid composition

The fatty acid profiles of the analysed cheeses are shown in Table 4. The share of individual groups of fatty acids in their profile depended on the type of milk used in cheese production. The highest share of unsaturated (UFA) and polyunsaturated fatty acids (PUFA) was determined in the cheese manufactured from ovine milk (B). This is typical for the fatty acid profile in milk fat of this species [39]. The obtained results were in agreement with those reached by Laskaridis et al. [40], who determined 27.33% of MUFA and 4.50% of PUFA in the fat of sheep’s milk feta cheese. The share of saturated fatty acids (SFA) and UFA in caprine cheeses (A and C) was not significantly different. The determined values were in accordance with the results presented by Van Nieuwenhove et al. [34] concerning fresh rennet-curd caprine cheese, the fat of which contained 69.1% of SFA and 32.1% of UFA. Pajor et al. [41] also determined that the fat of goat’s milk ripened rennet-curd cheeses contained 71.4–73.4% SFA, 22.8–24.3% MUFA and 3.7–4.2% PUFA. It is important to point out that the content of PUFA in cheeses A, B and C was higher than in cheeses D, E and F. Therefore, it may be concluded that from a nutritional point of view, the analysed cheeses made from goat and sheep’s milk were characterised by better fatty acid composition.

**Table 4:** Fatty acid profile of cheeses.

Fatty acid [%]	A	B	C	D	E	F
**4;0**	2.24^a^ ± 0.05	3.75^b^ ± 0.04	2.28^a^^b^ ± 0.03	2.92^a^^b^ ± 0.04	3.41^a^^b^ ± 0.05	3.43^a^^b^ ± 0.08
**6;0**	2.81^d^ ± 0.06	2.95^d^ ± 0.08	2.43^c^ ± 0.05	2.12^a^ ± 0.02	2.25^a^^b^ ± 0.06	2.38^b^^c^ ± 0.06
**8;0**	3.42^b^ ± 0.01	2.45^a^^b^ ± 0.01	2.73^a^^b^ ± 0.04	1.19^a^ ± 0.00	1.32^a^^b^ ± 0.04	1.36^a^^b^ ± 0.05
**10;0**	11.90^b^ ± 0.21	6.23^a^^b^ ± 0.01	9.68^a^^b^ ± 0.10	2.79^a^ ± 0.01	3.01^a^^b^ ± 0.06	3.25^a^^b^ ± 0.10
**10;1**	0.20^a^^b^ ± 0.00	0.28^b^ ± 0.01	0.19^a^ ± 0.01	0.26^a^^b^ ± 0.01	0.25^a^^b^ ± 0.01	0.27^a^^b^ ± 0.02
**12;0**	4.44^e^ ± 0.02	3.77^d^ ± 0.05	3.77^d^ ± 0.06	3.08^a^ ± 0.02	3.26^b^ ± 0.03	3.53^c^ ± 0.06
**14;0**	9.81^a^ ± 0.10	11.11^b^ ± 0.15	10.94^b^ ± 0.02	11.55^c^ ± 0.10	11.79^c^ ± 0.04	12.16^d^ ± 0.08
**14;1**	0.25^a^^b^ ± 0.00	0.43^a^^b^ ± 0.01	0.23^a^ ± 0.00	0.90^b^ ± 0.01	0.77^a^^b^ ± 0.00	0.79^a^^b^ ± 0.01
**15;0**	0.63^a^ ± 0.01	0.91^a^^b^ ± 0.01	0.82^a^^b^ ± 0.01	1.09^b^ ± 0.01	1.04^a^^b^ ± 0.00	1.09^b^ ± 0.01
**16;0**	24.71^a^ ± 0.18	29.02^a^^b^ ± 0.21	28.09^a^^b^ ± 0.12	37.25^b^ ± 0.09	36.31^a^^b^ ± 0.28	35.54^a^^b^ ± 0.14
**16;1n-9**	0.73^d^^e^ ± 0.02	0.69^c^^d^ ± 0.02	0.76^e^ ± 0.01	0.63^b^^c^ ± 0.02	0.62^a^^b^ ± 0.00	0.56^a^ ± 0.05
**16;1n-7**	0.71^a^ ± 0.01	1.77^a^^b^ ± 0.05	0.85^a^^b^ ± 0.04	1.82^b^ ± 0.04	1.80^a^^b^ ± 0.05	1.68^a^^b^ ± 0.03
**17;0**	0.34^a^ ± 0.01	0.36^a^^b^ ± 0.01	0.60^b^ ± 0.01	0.45^a^^b^ ± 0.01	0.44^a^^b^ ± 0.00	0.42^a^^b^ ± 0.01
**17;1**	0.17^a^ ± 0.00	0.24^a^^b^ ± 0.02	0.37^b^ ± 0.02	0.31^a^^b^ ± 0.00	0.28^a^^b^ ± 0.00	0.26^a^^b^ ± 0.00
**18;0**	10.37^b^ ± 0.13	7.47^a^ ± 0.11	8.95^a^^b^ ± 0.04	9.26^a^^b^ ± 0.09	9.01^a^^b^ ± 0.07	9.10^a^^b^ ± 0.03
**18;1n-9**	21.04^a^^b^ ± 0.20	16.86^a^ ± 0.15	21.94^b^ ± 0.02	20.01^a^^b^ ± 0.11	20.00^a^^b^ ± 0.03	19.37^a^^b^ ± 0.29
**18;1n-7**	2.49^a^^b^ ± 0.06	5.96^b^ ± 0.04	1.72^a^ ± 0.06	1.91^a^^b^ ± 0.02	1.90^a^^b^ ± 0.04	2.09^a^^b^ ± 0.07
**18;2n-6**	2.12^b^ ± 0.03	1.81^a^^b^ ± 0.03	1.97^a^^b^ ± 0.01	1.30^a^ ± 0.01	1.42^a^^b^ ± 0.03	1.47^a^^b^ ± 0.01
**18;3n-6**	0.07^a^^b^ ± 0.00	0.04^a^ ± 0.00	0.06^a^^b^ ± 0.00	0.08^b^ ± 0.00	0.06^a^^b^ ± 0.01	0.06^a^^b^ ± 0.01
**18;3n-3**	0.66^a^^b^ ± 0.03	1.26^b^ ± 0.02	1.09^a^^b^ ± 0.01	0.51^a^ ± 0.01	0.50^a^ ± 0.02	0.55^a^^b^ ± 0.01
**CLA**	0.69^b^ ± 0.01	2.30^c^ ± 0.04	0.37^a^ ± 0.04	0.37^a^ ± 0.01	0.35^a^ ± 0.01	0.41^a^ ± 0.01
**20;0**	0.14^c^ ± 0.01	0.14^c^ ± 0.01	0.11^a^ ± 0.01	0.11^a^^b^ ± 0.01	0.11^a^^b^ ± 0.01	0.13^b^^c^ ± 0.00
**20;1**	0.03^a^^b^ ± 0.01	0.13^b^ ± 0.01	0.02^a^ ± 0.00	0.07^a^^b^ ± 0.01	0.07^a^^b^ ± 0.01	0.08^a^^b^ ± 0.01
**SFA**	70.80^a^^b^ ± 0.07	68.15^a^ ± 0.12	70.39^a^^b^ ± 0.09	71.80^a^^b^ ± 0.03	71.94^a^^b^ ± 0.01	72.37^b^ ± 0.15
**UFA**	29.16^a^^b^ ± 0.07	31.77^b^ ± 0.12	29.55^a^^b^ ± 0.08	28.16^a^^b^ ± 0.03	28.02^a^^b^ ± 0.01	27.59^a^ ± 0.15
**MUFA**	25.62^b^ ± 0.07	26.36^d^ ± 0.07	26.07^c^^d^ ± 0.06	25.90^b^^c^ ± 0.03	25.69^b^ ± 0.01	25.11^a^ ± 0.16
**PUFA**	3.54^a^^b^ ± 0.01	5.41^b^ ± 0.05	3.48^a^^b^ ± 0.02	2.26^a^^b^ ± 0.00	2.33^a^ ± 0.02	2.48^a^^b^ ± 0.01

$\bar{x}$ ± sd; mean values ± standard deviation

a-e–statistically significant differences between means (*p*≤0.05) are marked by different letters in rows

Caprine milk fat is characterised by higher nutritional value and better digestibility in comparison to cow’s milk fat. This is due to a smaller diameter of fat globules, as well as a higher proportion of low molecular weight fatty acids. Additionally, these are more likely to be incorporated into triglycerides [36]. According to Park [9], a higher digestibility of goat’s milk, compared to cow’s milk, can be related to natural homogenisation of goat’s milk fat. Smaller fat globules, which are characteristic of goat’s milk, have a greater surface area. Therefore, lipases have better conditions during the digestion process.

The highest content of caprylic (C8:0) and capric (C10:0) acids was found in cheeses A and C. Milk from small ruminants, especially goat’s milk fat, contains a significant share of low- and medium-chain fatty acids. The sum of caproic (C6:0), caprylic (C8:0) and capric (C10:0) acid contents equals approximately 15% in caprine milk fat, whereas in cow’s milk fat–only 6% [8]. According to Chilliard et al. [42], the amount of C6-C10 fatty acids is at least 2-fold higher in goat than in cow’s milk. This is worth noting because it is the reason why goat’s milk and their products are characterised by a unique, tangy flavour. Moreover, goat’s milk, and cheeses made from it, contain a higher proportion of caproic and caprylic acids in comparison to cow and sheep cheeses, and the highest content of capric acid among all the mentioned products. Queiroga et al. [20] analysed Coalho cheese made from cow, goat as well as cow and goat’s milks mixed at a 1:1 ratio. They stated that the content of short- and medium-chain fatty acids, such as those caproic, caprylic and capric, was higher in cheeses made from or with goat’s milk.

The cheeses made from cow’s milk analysed in this study presented a higher content of palmitic acid (C16:0), which was in agreement with data presented by Queiroga et al. [20], Lucas et al. [43] and Sanz-Ceballos et al. [13]. The highest content of CLA was determined in cheese B. This might be closely related to the large amounts of this desirable health-promoting component in sheep’s milk. Sheep’s milk is known for its high content of CLA. Moreover, Pakulski et al. [40] investigated the fatty acid composition of sheep’s milk and 5 types of sheep’s milk cheese (curd, soft, brine, scalded-smoked and semi-hard cheese). They concluded that the highest content of CLA (0.92 g/100g) was reached when sheep’s milk was processed into scalded-smoked cheeses.

The fat content and fatty acid profile of milk is closely related to various factors such as those genetic, physiological and environmental, as well as nutritional [44]. The fatty acid profile in milk is strongly affected by the season due to changes in the forage composition of animals grazing on pastures [3, 45]. The differences among the share of particular fatty acids in their profiles between cheeses made from milk of different animal species can also be explained by different physiological regulation of the mammary glands. These regulations significantly affect the elongation process of fatty acids, which are synthesized by the fatty acid synthesis complex [43].

### Free fatty acid content

The free volatile fatty acid content in cheeses is shown in Table 5. The most abundant free fatty acid in analysed cheeses was acetic. This was in agreement with results achieved by Majcher et al. [46], who drew the same conclusion regarding *oscypek*, a raw sheep’s milk smoked pasta filata cheese. It is important to emphasize a significant difference in free acetic acid content between D (306.10 mg/kg) and F (55.49 mg/kg). It occurred despite the fact that D and F were made from pasteurised cow’s milk. This may indicate the occurrence of differences in the production process and the smoking of both cheeses. The content of butyric and caproic free fatty acids was several times higher in goat’s milk cheeses than in the other analysed samples. According to Bontinis et al. [47], raw goat’s milk cheese contains 71.0 ± 1.95 and 75.4 ± 2.45 mg/kg of free butyric and caproic acids at the beginning of ripening, respectively. A similar amount of butyric acid was determined in samples A and C.

**Table 5:** Free volatile fatty acid content in cheeses.

FVFA [mg / kg]	A	B	C	D	E	F
**Acetic**	138.57^c^ ± 8.99	187.75^e^ ± 2.23	165.78^d^ ± 10.84	306.10^f^ ± 2.17	110.99^b^ ± 1.68	55.49^a^ ± 1.87
**Propionic**	3.86^a^^b^ ± 0.29	2.14^a^ ± 0.04	4.09^a^^b^ ± 0.20	7.05^b^ ± 0.07	3.89^a^^b^ ± 0.06	3.44^a^^b^ ± 0.11
**Butyric**	78.43^b^ ± 4.89	11.18^a^^b^ ± 0.36	78.83^b^ ± 7.49	6.53^a^ ± 0.37	11.78^a^^b^ ± 0.84	11.73^a^^b^ ± 0.21
**Isovaleric**	8.79^c^ ± 0.99	1.94^a^ ± 0.27	6.42^b^ ± 0.01	10.47^d^ ± 0.27	5.58^b^ ± 0.01	2.83^a^ ± 0.12
**Valeric**	1.17^e^ ± 0.10	0.53^a^^b^ ± 0.01	0.87^d^ ± 0.14	0.85^d^ ± 0.08	0.72^b^^c^ ± 0.11	0.43^a^ ± 0.01
**Caproic**	47.70^b^ ± 1.47	4.61^a^ ± 0.26	45.03^b^ ± 2.66	3.33^a^ ± 0.05	5.72^a^ ± 0.08	5.05^a^ ± 0.27

$\bar{x}$ ± sd; mean values ± standard deviation

a-f–statistically significant differences between means (*p*≤0.05) are marked by different letters in rows

Raw milk and associated microorganisms (especially psychrotrophic bacteria) can be the sources of lipases. A lot of these enzymes are thermolabile. Therefore, they play an important role, mostly during raw milk cheese production [48]. Cheeses A and C, which contained the highest content of butyric and caproic free fatty acids, were produced from raw goat’s milk. As a result, a large amount of these fatty acids could have been released during lipolysis. Cheese B was also produced using raw milk. However, it was of pasta filata type, meaning the cheese curd is heated and pressed during production to achieve appropriate consistency and shape. Consequently, lipolytic enzymes could have been partially inactivated during this step.

### Cheese texture

The results of instrumental texture characteristics regarding the analysed cheeses are shown in Table 6 and a sample graph is presented in S2 Fig. The greatest hardness was determined in cow’s milk cheeses (approximately 10,000 G), followed by ovine cheese (about 6,000 G) and caprine ones (approximately 2,500 G). In general, the structure of casein micelles in cow, goat and sheep’s milk is similar. Still, significant differences in micelle size have been reported. The main casein micelle fractions (α_s1_-, α_s2_-, β- and κ-caseins) in goat and sheep’s milk are similar to those in cow’s milk. Nevertheless, it has been reported that size and ratio of these fractions differ depending on the species. While the main α_s_-casein fraction in bovine milk micelles is α_s1_-casein, goat’s milk micelles have a low concentration of α_s1_-casein. Moreover, in contrast to other small ruminant milks, the dominating casein fraction in goat’s milk is β-casein [9, 49]. Therefore, a lower total casein content, different ratio of casein fractions and relatively larger casein micelles explain the weak texture of gels and, consequently, the lowest hardness value of cheeses made from goat’s milk [8].

**Table 6:** Cheese texture.

Property	A	B	C	D	E	F
**Hardness [G]**	2892.2^b^ ± 216.7	5969.9^c^ ± 583.3	21388.7^d^ ± 2545.8	10561.0^a^ ± 157.9	9896.5^a^ ± 10.6	9186.8^a^ ± 218.5
**Adhesiveness [kG×;s]**	-94.83^a^ ± 91.62	-18.48^a^ ± 3.55	-0.89^a^ ± 0.23	-10.01^a^ ± 0.49	-350.43^b^ ± 85.25	-258.90^b^ ± 42.71
**Springiness [–]**	0.683^a^^b^ ± 0.078	0.597^a^ ± 0.065	0.950^c^ ± 0.010	0.710^a^^b^ ± 0.078	0.707^a^^b^ ± 0.012	0.773^b^ ± 0.006
**Cohesiveness [–]**	0.337^a^ ± 0.012	0.197^c^ ± 0.012	0.320^a^ ± 0.052	0.443^d^ ± 0.012	0.573^b^ ± 0.006	0.603^b^ ± 0.012
**Chewiness [G]**	663.65^b^ ± 22.15	699.65^b^ ± 133.83	6422.10^c^ ± 1072.10	3112.69^a^ ± 731.28	3949.45^a^ ± 32.81	4259.67^a^ ± 180.05
**Resilience [–]**	0.107^c^ ± 0.006	0.067^b^ ± 0.006	0.180^a^ ± 0.026	0.167^a^ ± 0.006	0.230^d^ ± 0.000	0.273^e^ ± 0.006

$\bar{x}$ ± sd; mean values ± standard deviation

a-e–statistically significant differences between means (*p*≤0.05) are marked by different letters in rows

Significant differences were also observed in chewiness. A and B were characterised by the lowest value of this feature (below 700 G). The highest value of chewiness was determined in C (approximately 6,500 G). The coagulation properties of cow’s milk have been extensively investigated by many authors, less so in sheep and goat’s milk. Calvo [50] compared rennet coagulation duration of cow, goat and sheep’s milk, concluding that the enzymatic phase proceeds faster in cow’s milk than in those from goats and sheep. Moreover, there was no significant difference between goat and sheep’s milk.

### Organoleptic quality of cheeses

**Fig 2:**
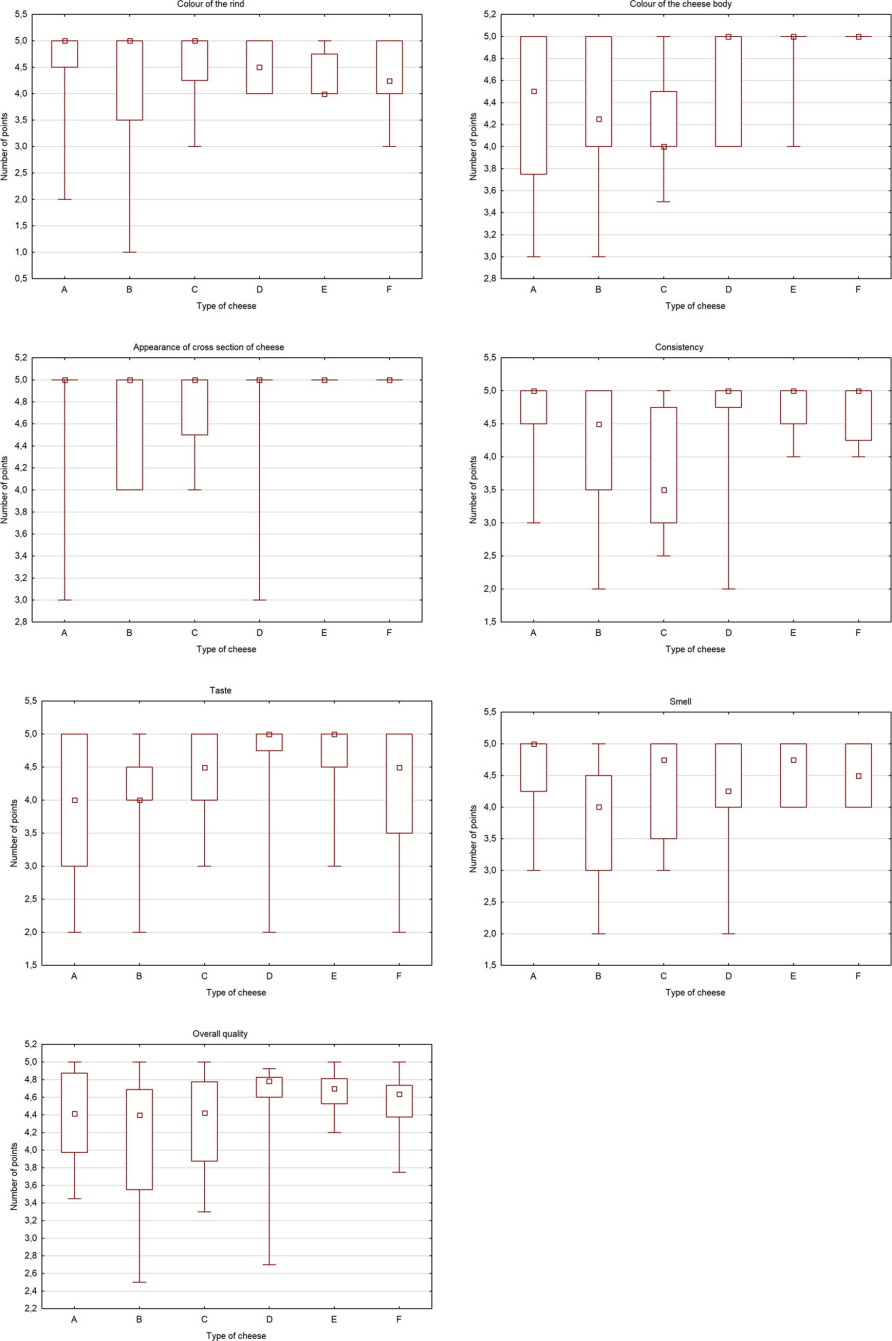
Organoleptic quality of smoked cheeses.

The results of organoleptic evaluation are presented in Fig 2. A square represents the median, a rectangle represents quartiles (25–75%), and line segments represent maximum and minimum values.

Analysis, performed using a 5-point scale showed no significant differences between the examined cheeses. Organoleptic quality of cheeses produced at small private farms was as good as those manufactured in industrial conditions. The median of the results with regard to the overall quality of D, E and F was between 4.6–4.8 and about 4.4 regarding the A, B and C cheese samples.

The sensory characteristics of cheese depend on many factors, including the type of milk, quality and composition of the raw material, as well as technology of production. However, the use of different types of milk and the differences found in the chemical composition of the tested cheeses did not reflect the results of organoleptic evaluation. This could have been caused by smoking. The smoking process influences, e.g. sensory characteristics. Natural smoke contains many odour-active substances, i.e. phenolic compounds, which play an important role during smoking. They exhibit a typical sharp and smoky smell, and, due to their very low odour thresholds (≤1 ng/cm^3^), they influence the sensory characteristics of smoked products [46].

According to Spiel et al. [14], with reference to goat cheeses only, young consumers most often prefer the smoked variety, followed by soft cheese, cottage cheese, processed cheese, and mould-ripened cheese. Moreover, Fangmeier et al. [35], analysed 6 cream cheeses made from cow, goat and buffalo’s milk and their mixtures. Respondents most frequently claimed that they would like to buy the cheeses made exclusively from cow’s milk. Organoleptic characteristics of caprine cheeses are an important factor in the marketability of these products and their consumer acceptability. Cheeses made from cow’s milk tend to have a more desirable taste, smell and texture than goat’s milk cheeses, especially after storage. The less desirable characteristics may be caused by the poor firmness of the curd and the specific taste, as well as smell [21]. However, this has not been confirmed in the present study. The obtained results show that the examined caprine, ovine and bovine milk cheeses were characterised by similar organoleptic quality. Therefore, it can be concluded that natural smoking may be used to increase the organoleptic quality of goat’s milk cheeses. Furthermore, according to our previous work, all the examined cheeses were characterised by a low amount of biogenic amines and polycyclic aromatic hydrocarbons–benzo[a]pyrene and the sum of benz[a]anthracene, chrysene, benzo[b]fluoranthene as well as benzo[a]pyrene [51].

## Conclusions

The nutritional value of the cheeses depends on the type of milk used in their production. Small ruminant milk cheeses were characterised by a higher PUFA content compared to those from cow’s milk. Moreover, cheese made from sheep’s milk was characterised by the highest CLA content, as well as the highest share of UFA and PUFA. The highest content of butyric and caproic free fatty acids was determined in goat’s milk cheeses. Texture analysis allowed to demonstrate that the hardness of cheeses made from the milk of various animal species differed significantly. Organoleptic assessment did not reveal any significant differences between cheeses, regardless of the type of milk from which they were produced. Moreover, organoleptic quality of cheeses produced at small private farms was as good as those manufactured in industrial conditions. Taking the above into account, it may be concluded that the profile of fatty acids, amount of FFA, as well as texture, are not the main factors influencing the organoleptic evaluation of smoked cheeses, the results of which do not indicate significant differences in any of the analysed quality determinants ors the overall acceptability of the cheeses. Therefore, the application of smoking has a positive effect on the organoleptic quality of goat and sheep cheeses, the taste and smell of which are usually not acceptable to some consumers.

## Supporting information

S1 Fig Sample chromatogram showing amino acid profile in cheese. (TIF)

S2 Fig Sample graph showing texture profile of cheese. (TIF)
