# Supplementary figures and images for: Nutritional value and organoleptic assessment of traditionally smoked cheeses made from goat, sheep and cow’s milk
Source: PLoS One. 2021 Jul 22;16(7):e0254431. doi: 10.1371/journal.pone.0254431 (PMC8297890; doi:10.1371/journal.pone.0254431)

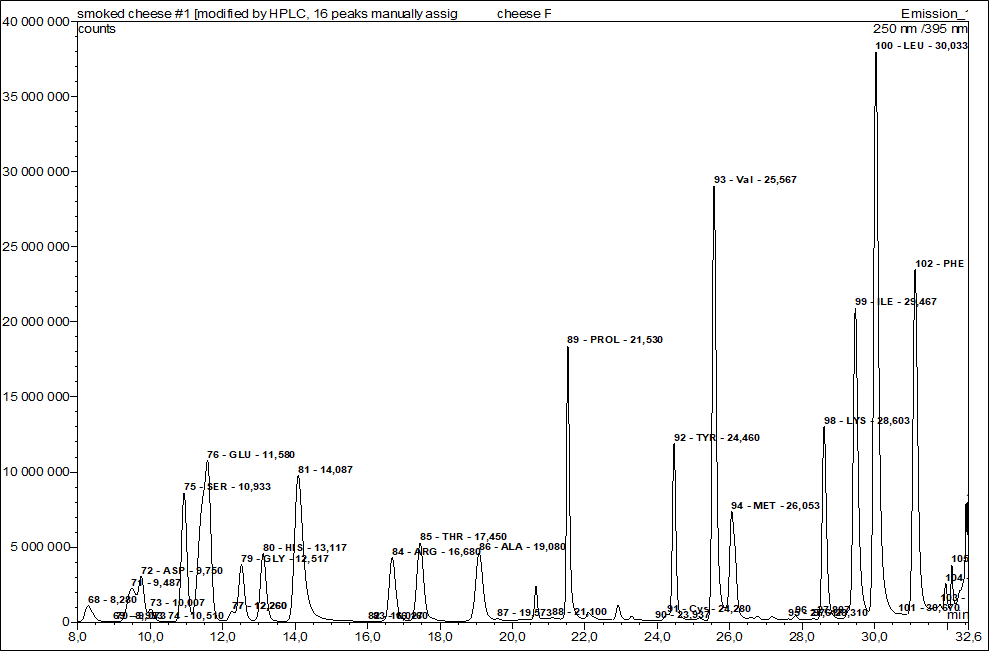

Supplement: S1 Fig — (TIF) [file pone.0254431.s001.tif]

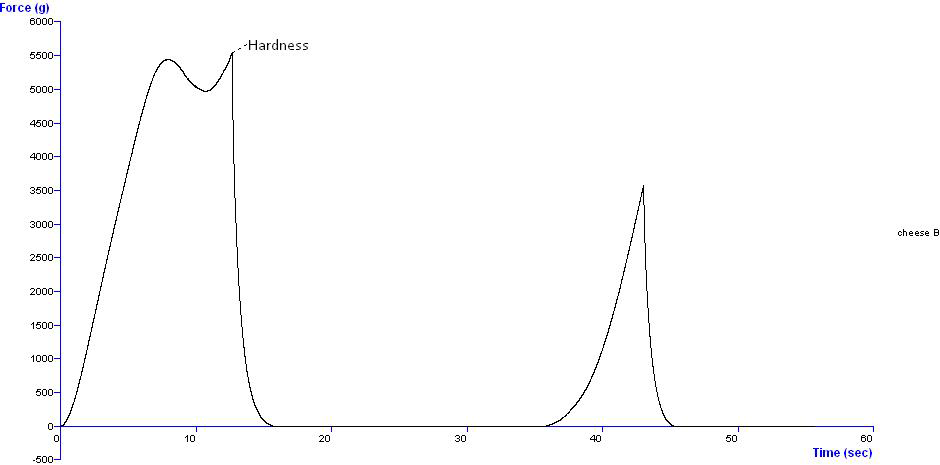

Supplement: S2 Fig — (TIF) [file pone.0254431.s002.tif]
